# Conversation analysis, institutions, and rituals

**DOI:** 10.3389/fsoc.2023.1146448

**Published:** 2023-10-10

**Authors:** Pertti Alasuutari

**Affiliations:** Tampere University, Tampere, Finland

**Keywords:** conversation analysis, new institutionalism, sociological institutionalism, new materialism, ritual theory

## Abstract

By relating conversation analysis (CA), in particular CA research on institutional interaction to such research traditions as sociological institutionalism, new materialism, and ritual theory, the article illustrates how CA scholarship can contribute to macrosociological theorizing. This argument is illustrated by how national parliaments are organized as institutions. The main point made in the article is that occasions of what CA calls institutional interaction should be considered as rituals. Although those occasions are scripted ceremonial performances wherein social pressure, material conditions, or avoidance of punishment make actors conform, they still play a role in constituting social order by making participants honor the rules and principles codified in an organization’s frontstage events. The article also underlines that organizational arrangements do not determine what actors can say or do, but they impose limits and conditions on people’s conduct. Finally, the paper suggests that it is through such arrangements of institutional interaction that social structure is created, maintained, and naturalized.

## Introduction

Because of its roots in ethnomethodology, conversation analysts have avoided building macrosociological theories. Using concepts that define society or its constitutive elements would violate the principle that an ethnomethodologist should analyze the methods and concepts members of a community use to produce social order in and through social interaction (Garfinkel, 1964; Garfinkel, 1967). However, conversation analysis (CA) has not entirely succeeded in staying clear of all references to society at large. After all, a considerable and most intriguing part of CA research is called research on institutional interaction, which implicitly acknowledges that there are objects called institutions out there. I suggest that, when related to other research on institutions, the insights gained from this scholarship can bridge the gap between CA and macrosociological theory. In this paper I suggest how that can be done, and hence contribute to the discussion on how conversation analysis could extend its scope toward addressing macrosociological questions.

CA’s interest in institutions is a good starting point, because institution is one of the conceptual tools by which social theorists have tried to answer a fundamental question of sociology: how is social order possible? CA suggests that the basic answer is the interaction order: rather than members of society observing pregiven, internalized norms or meanings, social order is always negotiated in interaction situations. What CA calls institutional interaction is an extension to the basic answer: in institutional settings participants negotiate social order under special conditions and restrictions in comparison to the features of ordinary conversation between peers. This has been shown by studying interaction in, for instance, courtrooms, classrooms, interviews, therapeutic sessions, and different technical settings (e.g., Drew and Heritage, 1992; Arminen, 2005; Arminen et al., 2010; Ekstrom, 2012; Ilomäki and Ruusuvuori, 2022).

This raises the question, how do those institutional settings come about? It would be far too voluntaristic to claim that interactants create those settings on the spot by deciding to assume roles such as judge, prosecutor, and defendant. Since institutions are not God-given but instead designed by people, clearly their constitution and proliferation are another element in the creation and maintenance of social order. Therefore, in this article I aim to show that it is fruitful to complement CA with institutional theory, which primarily focuses on studying how various organizations—private companies, state bureaucracies, and non-profit organizations—structure and manage the social world. Although neoinstitutional scholarship studies various organizations and identifies their specific features, it shares with CA the view that there is something generic about all institutional interaction—or I could phrase it organizational behavior—regardless of what organizations we are talking about. By utilizing the neoinstitutionalist insights about organizations and organizational behavior, CA scholarship can make itself relevant at a macrosociological level.

What I mean by the neoinstitutionalist insights is that organizations are what I would call designed institutions. That is, in their conduct actors are expected to observe inscribed rules and principles, which often leads into ceremonial behavior (Meyer and Rowan, 1977) and to a division between frontstage and backstage settings (see, e.g., Goffman, 1955, 1956). In many instances this appears to suggest that ceremony has little impact on people’s lives: on formal occasions actors pay lip service to high principles but act according to informal rules to get things done in practice. Yet I suggest that formal occasions are also important for social order: they are rituals that still honor and contribute to sanctifying the rules and principles in question. From this perspective, organizations and organizational behavior are key to the creation and maintenance of social order.

To elaborate on what I mean by that, I will lead you through a discussion of how CA research on institutional interaction relates to sociological institutionalism, new materialism, and ritual theory. To illustrate the points made, I discuss the way parliamentary politics is organized throughout the world.

### CA, institutions, and organizations

In his article on conversation analysis as social theory, Heritage (2009) concludes that CA’s main input to social theory is in pointing out how the interaction order—that is, ordinary conversation—is managed as an institution in its own right. But what other institutions are there? Sociology textbooks include longer or shorter lists of other institutions, but—perhaps except for language in the sense that humans can create shared sign systems—I suggest there is a difference between ordinary conversation and other institutions. To put it shortly, conversation is a universal institution, but others are culture-specific, historical formations.

To elaborate on that point, it is certainly possible to study conversation as an institution from a historical perspective and, respectively, to try and find universal features in, say, all religions of the world as Durkheim (1995) did. In that respect, CA scholars’ long-term program to identify and describe the basic organizational principles found in all conversations is a choice of perspective. CA researchers consider universal the patterns or basic elements of the interaction order, termed by concepts such as adjacency pairs. They have indeed shown convincingly that the basic sequence organization of ordinary conversation is followed everywhere in the world regardless of local language and culture.

What about the others? Social and cultural theorists have argued that, for example, religion is an institution that can be found in all human societies. However, I tend to agree with the scholars who claim that the unifying features of present-day established religions are due to religious organizations copying models from one another, and that the whole concept of religion that lumps them together is problematic (Taira, 2010; Taira, 2022). The same goes for other candidates in the lists of social institutions: family, law, education, economy, etc. Functionalist theories of society have tried to define a list of key institutions that any society has to have to function and further develop (see, e.g., Parsons, 1951, 1964, 1966), but this line of thought can be challenged by the so-called Galton’s problem, according to which such similarities between societies as similar organizational structures, policies, and socioeconomic development can be result of diffusion or borrowing among them (Ross and Homer, 1976; Braun and Gilardi, 2006). There is, indeed, plenty of evidence of worldwide emulation between organizations, both within particular categories of organizational activities such as lawmaking (Watson, 1974; Twining, 2004) and across the entire field of all kinds of organizations (Meyer and Bromley, 2013; Bromley and Meyer, 2015).

CA scholarship shares the scepticism or cautiousness of new institutionalism in listing universal institutions, although within CA research there is plenty of research on interaction in different institutional contexts. Following the methodological principle of using the concepts “members” use to refer to social phenomena only as a topic, not as a resource (Garfinkel, 1964; Zimmerman and Pollner, 1970), CA researchers have not referred to these contexts as separate institutions. Instead, they have avoided the question by lumping all these settings studied under the term “institutional interaction.”^1^

What CA scholars imply by talking about interaction in “institutional settings” is that in other than ordinary conversations participants are expected or in varying ways forced to follow rules that put constraints on the forms and content of their interaction. To phrase this by using Franco Ferraris’ concept, participants follow inscribed rules, “characterized by being written on a piece of paper, a computer file, or at least in the heads of the people involved” (Ferraris, 2013: 4). As a small correction to Ferraris’ definition: the rules observed are not necessarily known to all participants: “clients” entering a professional’s appointment, a courtroom or, say, a computerized online service platform, do not necessarily know how they are expected to behave. They are directed or punished if they violate the rules or deviating from their expected role is made technically impossible.

By talking about inscribed rules and instructions guiding actors’ conduct, I underline the point that in these contexts, people’s behavior, social and spatial positions, and mutual interaction are consciously designed and organized by inscribed regulations or norms, often also by material structures such as the built environment. They are designed institutions. Latour’s (1994) discussion of speed bumps as actors guiding traffic behavior is a fine reminder; the ways our activities are channeled in the online systems is another one. When we think about contemporary society at large, this is it: we live amid a massive, carefully designed and built configuration of institutional arrangements that guide our conduct and constitute reality for us.

The point that institutions are designed by humans does not mean that written regulations aimed at steering people’s behavior come first. Rather, technical inventions often open new possibilities for people’s activities, which then give rise to regulations. In this respect, the invention of money in the modern sense somewhere around 7th century BC (Weatherford, 1997) is the starting point for and a connecting link between various present-day institutions. When we think about institutions such as doctor-patient interaction and various other encounters involving monetary transaction between buyer and seller of products or services, they are constituted by money as the medium. Monetary economy then enables the formation of various occupations and professions and creates need for laws and regulations.

Clearly, then, talk about institutions leads to a discussion about the entire modern society and world system which, from this perspective, is composed of various interlaced, historically evolved institutions. There is cultural uniqueness and variation in the functioning of various institutions in different parts of the world, but structural isomorphism (that is, you find the same organizations such as a government, ministries, and universities with similar features and sub-structures in each national state as a component part of world society) is amazingly big, considering the vast differences in material resources between countries (Meyer et al., 1997). Monetary economy of course ties the world into a single place, but the political organization wherein the entire globe is composed of formally sovereign nation-states also contributes to considerable likeness.

### Sociological institutionalism and organizational behavior

While CA research on institutional interaction has paid attention to the procedural rules and limitations that impose formal constraints on people’s talk and behavior, neoinstitutionalist scholarship has also underlined actors’ ceremonial behavior in organizations. The perspective is, however, quite different, because scholars in this field focus not only on face-to-face interaction but on how organizations are managed, what structures and substructures are instituted, and how the organization presents itself to its peers and to the outside world. According to the seminal article by Meyer and Rowan (1977), ceremonial behavior stems from the fact that an organization needs to adopt all kinds of standards and practices instituted in the organizational field where it is situated, but conformity to institutionalized rules often conflicts sharply with efficiency criteria. Paradoxically, such externally legitimated standards are promoted and justified by rationality and efficiency. To maintain ceremonial conformity, organizations tend to buffer their formal structures from the uncertainties of technical activities by becoming loosely coupled. Therefore, gaps emerge between their formal structures and actual work activities.

A sick worker must be treated by a doctor using accepted medical procedures; whether the worker is treated effectively is less important. A bus company must service required routes whether or not there are many passengers. A university must maintain appropriate departments independently of the departments' enrollments. Activity, that is, has ritual significance: it maintains appearances and validates an organization (Meyer and Rowan, 1977: 355).

In addition to decoupling between formal structures or mission statements and actual practices, reflected in ceremonial behavior and hypocrisy, Meyer and Rowan point out that conformism results in growing isomorphism: organizations imitate one another within and between different organizational fields. To list examples given by Meyer and Bromley (2013: 367), hospitals and medical practices, religious congregations, recreational programs, traditional charities (now “nonprofit organizations”), and universities around the world become similarly managed organizational actors. Simultaneously state bureaucracies are pressed by policy advice organizations to become accountable, purposive, decision-making organizations.

Applying the same basic ideas to the entire global system, neoinstitutionalist world society scholarship shows that the entire world system is composed of isomorphic building blocks such as national states and organizations, built by applying the same worldwide models. These models “define and legitimate agendas for local action, shaping the structures and policies of nation-states and other national and local actors in virtually all of the domains of rationalized social life—business, politics, education, medicine, science, even the family and religion” (1997: 145).

### Construction of the social world

To fully realize what all this conformism and resultant isomorphism means, we need to think about it from a sociology of knowledge perspective. It is the structural isomorphism between organizations that makes it possible for us to identify and categorize different kinds of organizations: schools, religious congregations, private enterprises, state bureaucracies. It is easy to take it for granted that such organizational types resemble one another because of the functions they serve in society. Organizations can be likened to different plants growing in the nature, which assume their shape and special properties through natural selection as adaptations to their ecological niche: climate, soil, and competitors. But organizations do not evolve through a natural, evolutionary process because they are designed institutions, in each case established and modified by people, whose beliefs about efficient and well-managed organizations their formal rules and structures reflect. The creators may be intelligent, but not in the sense that proponents of intelligent design have in mind: organizations are made by humans.

This brings to the fore the point that ideas matter in erecting organizations. They are never built from scratch but instead, when people establish a new business, association or, say, a religious congregation, they study how others have done it and how successful they have been. Besides, for us to establish an organization belonging to a particular category, we already need to have a general idea of what that means. Furthermore, there are laws, regulations, and recommendations about how an organization should or must be established and managed.

Consider the Finnish Associations Act. It states that an association may be founded for the common realization of a non-profit purpose, which may not be contrary to law or proper behavior. Section 7 states:

A charter shall be drawn up on the founding of an association and the rules of the association shall be annexed thereto. The charter shall be dated and be signed by three or more persons joining the association. A natural person as a founder shall be 15 years of age or over (Finnish Patent and Registration Office, 2022).

The law text then goes on determining how joining, resigning, and expulsion from an association is done, how decisions are made, meetings organized, and matters to be decided in meetings. Because of such rules, it is no wonder that there is structural isomorphism between associations.

These provisions are stated in the Finnish law, but even if a national law does not regulate life in associations so meticulously, there are national and international recommendations and standards that promote good practices. Consider the way formal meetings are organized. In the United States and other English-speaking countries, actors observe Robert’s Rules of Order, a manual of parliamentary procedure by U.S. Army officer Henry Martyn Robert, first published in 1876 as an adaptation of the rules and practice of the United States Congress to the needs of non-legislative societies. Very similar manuals about the rules observed in formal meetings in *ad hoc* instances, associations, legislatures, and business organizations can be found throughout the world.

One might argue that democratic organizations are similar throughout the world because people like Henry Martyn Robert have taken the effort to analyze and crystallize the rules people intuitively follow in democratic meetings. Therefore, such work could be likened to linguists writing the grammar of a language: in doing that linguists do not order how people should talk correctly, they only record the logic of that language. But regardless of the historical origins of the first rules of order for democratic meetings, present-day organizations have copied them from codified rules.

For ideas to effectively spread from one organization to others, people need to describe and define them at a more general level. In that sense, what Strang and Meyer (1993) call “theorization” is a key institutional condition for diffusion. That is, for a practice such as Japanese “quality circles” adopted in several factories to spread effectively to different countries, it needs to be formulated at a rather general level as a universal model, detached from contexts in which it is first employed, or which serve as food for thought in creating the model. Therefore, Strang and Meyer note, scientists and policy experts serve a role in constructing models that are assumed to be universally applicable.

Empirical research on the formation and spread of worldwide models shows that the processes are quite complex. Policies are not packages that fly around and stick to organizations. Instead, following Latour’s (1986) suggestion (see also Callon, 1986), Czarniawska and Sevón (1996) prefer to talk about a process of translation, in which humans have an active role in circulating and shaping ideas. Adopting an exogenous model in an organization typically triggers a process of domestication, which results in adapting the model to the local conditions (Alasuutari and Qadir, 2014). Furthermore, rather than being first formulated and then spread, models are formed in parallel with their diffusion, and naming them is a key part of the practices through which they are promoted (Syväterä and Qadir, 2015). Typically, once there is a handful of organizations that have instituted a similar practice or organ, the representatives of the new institutions form an international organization that starts to brand and codify the model and recommend it to the rest of the world (Alasuutari, 2016).

This means that designing organizational practices and theorizing about them plays a central part in constructing the social world. Legislators, lawyers, economists, and social scientists design institutions, collect information about the existing ones, theorize about their functioning, and problems therein. Consequently, society with its various institutions presents itself to us in terms of ready-made concepts such as association or religion.

## Ritual practices

As discussed above, behavior in formal institutions is often ceremonial. Because of externally imposed regulations or recommendations, participants follow procedures that have no other meaning than fulfilling the law or keeping up appearances (Meyer and Rowan, 1977). For example, in Finland parents and kindergarten teachers are expected to prepare and sign an individual early childhood education and care plan for each child every year, although those plans are rarely looked at during the year, and the expectation that each child in a group could follow their individual education plan is unrealistic (Alasuutari and Alasuutari, 2012; Alasuutari et al., 2014, 2020). From this perspective, ceremonial aspects of interaction in formal institutions are consequence of the need or willingness to copy exogenous models that remain mere formalities, meaningless or harmful for actual business. But organizational practices assume formal, invariant patterns also for other reasons. Some events are designed to be ceremonial in the first place. Rather than hollow formalities that endanger the legitimacy of the institution, rituals organized in an institution are meant to grant it extra legitimacy and sacredness.

Interestingly, to create the feeling of a special, emotionally touching event, designers of rituals resort to similar techniques that characterize interaction in institutional settings. CA scholars note that institutional contexts are manifested in, and in turn shape, the actions of both professional and lay participants, whose speaker roles and forms of talk may be carefully defined (Drew and Heritage, 1992; Arminen, 2005). Rituals are characterized similarly. According to Bloch (1989), ritual is an occasion where syntactic and other linguistic freedoms are reduced because ritual makes special uses of language: it is characteristically stylized speech and singing. This affects the contents of talk in rituals.

The formalization of speech therefore dramatically restricts what can be said, so the speech acts are either all alike or all of a kind and thus if this mode of communication is adopted there is hardly any choice of what can be said. Although the restrictions are seen usually as restrictions of form rather than of content, they are a far more effective way of restricting content than would be possible if content were attacked directly. Formalization therefore goes right through the linguistic range. It leads to a specially stylized form of communication: polite, respectful, holy, but from the point of view of the creativity potential of language, impoverished (Bloch, 1989: 27).

In similar vein, Bell (2009) defines rituals as occasions in which action is *formalized*, *rule-governed* and *invariant*. Furthermore, rituals are often meant as *performances*: spectacular, public events. Many rituals are also *traditionalistic* in that they reference an old tradition. They may celebrate a special occasion or mark a transition in members’ status. Rites of passage (van Gennep, 1960) such as graduations ceremonies, weddings and funerals are good examples (Meyers, 2016; Ozbolat, 2019).

Originators typically copy rituals or their elements from elsewhere. For example, the Soviet Union’s establishment created rituals for the same occasions as in other countries: birth, coming of age, wedding, funeral, and initiation into working life positions (Lane, 1981). In inventing rituals, designers are also eclectic: they copy elements and symbols that have been found impressive and sanctified in other rituals. Consider taking an oath in a court of law or in a parliament as a new member. Typically, the individual is expected to put their left hand on a Bible or some other book that the people in question consider sacred.

Rituals are an important aspect of actions in formal institutions because through them beliefs, emotions, and identities can be formed and changed, as Islam and Zyphur (2009: 114) note. Like the bulk of research and theorizing on ritual, they emphasize its symbolic character, which not only affects individuals but plays an important role in maintaining and reinforcing social structures and incorporating individuals into a larger social entity.

I would also emphasize the bodily and material aspects of rituals. In them, everything participants do may be carefully designed: whether they sit or stand; where different actor groups are situated; how and when they move; what and how each actor speaks or sings; and whether they eat, drink or smoke something during the ritual. The setting may also be specifically designed for the occasion. For example, the space may be heated, as is the case with the sweat lodge ceremony initially created by some of the indigenous peoples of the Americas (Wikipedia, 2022). It is also common that the room places leading actors to sit or stand higher up than the rest of the participants. The distances between different actor groups may also be determined by the architecture: for example, how the chairs for the audience are situated and in what rows different audience groups are placed. Furthermore, the space may be decorated by different items such as drawings, texts, emblems, flags, carpets, and other textiles.

The bodily, material, and symbolic aspects of rituals are naturally enmeshed. When people’s behavior is stylized, it can carry special meaning beyond the exact words spoken or the mundane significance of acting in peculiar ways like sitting, standing, kneeling, or making signs with one’s hands. The bodily engagement makes rituals holistic, also emotionally felt experiences, which helps creating associations between acts, their symbolic meaning, and the community in question.

There are rooms or entire buildings designed with the needs of rituals in mind only for institutions that are considered especially important: temples, parliament buildings, and courts of law are obvious examples. However, most ritual practices occur in ordinary built or natural environments. In most countries a priest or civil servant can officiate a wedding anywhere. Official statements, requests, agreements, and other inscribed acts typically require specified formulations, increasingly often enforced through a ready-made template or online form to be filled out. Such regulations make the acts official, legally binding, or otherwise acknowledged by a community or organization, but they also contribute to guiding the interaction and the discourses used in it to forms that differ from ordinary conversation between peers.

If not spectacular ritual performances, some activities in designed institutions can be considered rituals in that they comprise invariant practices: things must be done in particular ways to be considered legitimate, for the organization in question to accomplish its tasks. The material organization and the rules governing behavior are expected to ensure that the institution works efficiently toward its goals. How that is supposed to happen varies depending on the type of organization. It is assumed that in business companies and armies, every member works for the same goal, whereas it is thought that deliberative decision-making institutions such as legislatures and judiciaries work best when they can ensure free and open exchange of views that results in optimal choices.

But regardless of such differences, actors’ activities in institutional contexts cannot be deduced directly from organizations’ stated goals or organizational charts. At some level, many organizations have internal discussion, disagreements or even disputes about their aims and means, and members may compete with one another about their power positions and career development. From this viewpoint, the settings and regulations created for an organization can be likened to the rules of a game (North, 1990). Once the conditions for activity within an organization have been defined, actors start inventing strategies by which to play the game to advance their goals. The formal rules are also complemented by principles honored in society at large. Therefore, an informal organization emerges as a refracted reflection of the official picture, supplementing, modifying, or challenging the formal rules. I suggest it is the interplay between formal and informal organization that constitutes an institution that we routinely refer to by its name. The way in which national legislatures function in the modern world is a prime example.

### Institutional construction of parliamentary politics

While social constructionism and sociological institutionalism want to unpack social orders, showing how they are historical formations, functionalist and rational-choice approaches consider many modern formal organizations as outcomes of an inevitable process of modernization, determined by pure reason. This is especially the case with such highly valued institutions as parliamentary democracy. The way national policy decisions have been prepared and made particularly in the British Parliament and other Western democracies has been hailed as an arrangement that is closest to a universal ideal of communicative action or deliberative rationality in which the best argument eventually wins (Rawls, 1971; Habermas, 1984). For example, Palonen (2019) builds an ideal type of deliberative parliamentary practice with Westminster as its historical approximation. In this respect he agrees with Jeremy Bentham, who already in the early 19th century was amazed at the realization that when he took as his task to define the rules that are necessary to every assembly, they turned out to be those very rules actually observed in both assemblies of the British Legislature (Bentham, 1843).

Yet, when we scrutinize the shaping of the modern notion of politics with the national parliament as its core institution, we can note that it evolved through emulation, coupled with an interplay between rules and tactics. Since several countries in Europe and later in other parts of the world had imitated one another in establishing national legislatures, tactics by which played the game also traveled between the parliaments. That way, the vocabulary, discourses, and procedures associated with what we understand by politics spread worldwide.

The systematic use of legislative obstruction tactics, first introduced in the British House of Commons in the latter half of the 19th century (Vieira, 2015: 84–123) is an example of how the rules of the game interact with the evolving tactics. To delay the legislative process, oppositional forces began posing questions to the Minister or giving lengthy speeches which were largely irrelevant to the topic at hand. This, in turn, led to the Parliament enacting new Standing Orders that limited opportunities for obstruction. And when there was growing dissatisfaction in the British Parliament in the latter half of the 19th century regarding the inefficiency with which the House of Commons passed laws, the same discourse spread throughout the British World and led to procedural reforms that mimicked those made in Westminster (Vieira, 2015: 124–173). And news about legislative obstruction in the Westminster also spread outside the English-speaking world. In Finland, Finnish-speaking nationalists in the estate Diet and socialists in the early unicameral parliament, Eduskunta, used the concept of parliamentary delay to accuse the old political elites of obstructing necessary reforms (Pekonen, 2017).

The rules observed in different institutional settings of the national parliament and how these settings relate to each other are another fine example. Since politicians’ work is to negotiate majority agreements about how a country is governed, one could well assume that the interests of different electorate groups and other stakeholders would be publicly discussed in the floor debates. That is not the case. Instead, the institutional settings wherein parliamentarians negotiate about decisions to be taken are divided into two contexts of interaction and bargaining, the public and the confidential. Goffman (1974) refers to a similar division into two institutional settings by the distinction between frontstage and backstage behavior. The floor sessions are public performances and legislatures also keep public record of their contents, whereas backstage bargaining is an only partially visible part of the work through which politicians negotiate public policy.

Formally, parliamentarians are independent actors invested with the power to represent their electorate and, as a collective actor, the sovereign nation, but behind the scenes they need to manage various kinds of interdependencies. The confidential contexts entail informal discussions and lobbying, negotiations in which actors are engaged, and the deals they make to form majority vote. The public contexts contain all the policy documents and floor debates in which proposals are promoted and decisions justified.

These two parts are in constant tension with each other. Speakers make references to the unscrupulous reality of politics happening behind the curtains where people seek their personal gain or group interest but present their own proposals as pure reason serving the whole nation or humankind. Yet public parliamentary discourse does not consist of mere rhetorical tricks by which politicians seeking partial interests make their aspirations and goals seem altruistic. In aspiring to persuade others, actors appeal to values and moral principles that not only guide and inform the views and identifications of their audience but also their own worldview. In any case, this tension between the two parts of legislative business shapes the forms of argumentation evident in national parliaments.

Talking about the backstage of policymaking does not mean that parliamentarians or the public are entirely unaware of agreements made behind the scenes. There are constant references to the background bargaining made within and between parties. For example, politicians are aware when members of a party in the government must vote for a decision against their “conscience” because of party discipline. In such cases, they are accused of compromising their personal integrity. Yet, because it reveals the government’s internal tensions, the opposition expects to see the government party or parties to agree on an issue amongst themselves and then stand behind a bill unanimously. Similarly, if it is well known that a legislature must take a decision because of external pressure, for instance to fulfill the criteria for getting a loan from the World Bank, parliamentarians consider it preferable that the negotiations are held behind closed doors. Making such coercion public in floor debates damages the public image of the national parliament as a sovereign institution.

The division into frontstage and backstage parts of legislative business is indeed evident in parliamentary practices and discourses in many ways. The fact that politics and politicians are often used as derogatory terms in the very institution dedicated to it stems from this same phenomenon. In the public, politicians aspire to defend their views as only informed by scientific evidence and by their altruistic goal to serve the nation, not the interests of any subgroup. In this discourse, others can be accused of “politicizing” an issue—that is, advancing their own interests. Referencing someone as a politician can in that sense be used as a derogatory term (Palonen, 2022).

## Conclusion

The task I set myself for this article was to point out how CA could extend its scope toward addressing macrosociological questions. To show CA’s relevance and links to some other schools of thought, I complemented CA research with institutional theory, especially sociological institutionalism, new materialism, and scholarship on ritual. Although I avoided unnecessary name dropping, it is obvious that the approach and lines of thought presented here also agree with, say, Berger and Luckmannn’s (1967) social constructionism and Bourdieu’s (1977, 1995) analyses of practices.

The main point I wanted to make was that occasions of what CA calls institutional interaction should be considered as rituals. Although those occasions are scripted ceremonial performances wherein social pressure, material conditions, or avoidance of punishment make actors conform, they still play a role in constituting social order by making participants honor the rules and principles codified in an organization’s frontstage events.

National parliament is a good example. Not to even mention authoritarian regimes, in many countries actual decisions are taken well before they are introduced and debated in plenary sessions. Yet in all legislatures of the world, in the frontstage occasions policies are justified (and criticized if that is allowed in a regime) by appealing to morally valid principles such as parliamentarians’ independence, national sovereignty, and the interest of the nation. Egotistic motives and deals between different groupings are not publicly disclosed. This could be seen as proof that frontstage rituals do not have any significance, but the point is that organizations such as national parliaments even in autocratic regimes still bother to put on the show. In other words, the social order is legitimated by the moral principles as conceptions of appropriateness.

Another point I wanted to make is that rituals are not so empty and meaningless as they may seem. They keep alive the values and principles that are honored and sanctified by them. In addition to moral principles, occasions of institutional interaction also construct and sanctify social positions, and hence the social order, in a very concrete manner. The arrangements of encounters in a designed institution place actors in positions that determine how must or can behave, for instance what options for tactics and resistance they have. Participants are made acutely aware of rituals as special occasions in a holistic manner that also entails their bodies, which strengthens the mental association between an occasion and what it stands for. For example, leaders are often placed higher up in the space, so that others must look up to them.

Third, the example of national parliament as a designed institution also illustrates the point that organizational arrangements do not determine what actors can say or do, but they impose limits and conditions on their conduct. Behavior is channeled to the possible modes, and to advance their views and objectives, people create various tactics by which to make use of or bend the rules of the game. When new tactics are invented, they spread to other similar organizations, which may create need for the organizations to renew their rules.

Finally, I suggest it is through such arrangements of institutional interaction—that is, ritual conduct—that what we call social structure is created, maintained, and naturalized. We are born to a world that presents itself through self-evident concepts, the built environment and artifacts, practices, conceptions of proper conduct, and identifications with various communities. As invariant performances and practices rituals also speak to our bodies and emotions, making us feel that, say, some things, principles, positions or persons are particularly important or even sacred.

## Author contributions

The author confirms being the sole contributor of this work and has approved it for publication.

## Footnotes

^1^By the way, since CA considers the interaction order as an institution in its own right, drawing a distinction between “ordinary conversation” and “institutional interaction” is a problematic solution: by definition, ordinary conversations are also an example of institutional interaction.

## References

[ref1] Alasuutari Pertti. (2016). The synchronization of National Policies: ethnography of the global tribe of moderns. London: Routledge.

[ref2] Alasuutari P. Alasuutari M. (2012). The domestication of early childhood education plans in Finland. Glob. Soc. Pol. 12, 129–148. doi: 10.1177/1468018112443684

[ref3] Alasuutari Maarit Markström Ann-Marie Vallberg-Roth Ann-Christine. (2014). Assessment and documentation in early childhood education. London: Routledge.

[ref4] Alasuutari Maarit Kelle Helga Knauf Helen. (2020). Documentation in institutional contexts of early childhood: normalisation, participation and professionalism. Wiesbaden: Springer Vieweg.

[ref5] Alasuutari Pertti Qadir Ali, (2014). National policy-making: domestication of global trends. London: Routledge.

[ref6] Arminen I. Auvinen P. Palukka H. (2010). Repairs as the last orderly provided defense of safety in aviation. J. Pragmat. 42, 443–465. doi: 10.1016/j.pragma.2009.06.015

[ref7] Arminen Ilkka. (2005). Institutional interaction: studies of talk at work. Aldershot, Hants, England; Burlington, VT: Ashgate Publications.

[ref8] Bell Catherine. (2009). Ritual: perspectives and dimensions. New York: Oxford University Press.

[ref9] Bentham Jeremy. (1843). "The works of Jeremy Bentham, Vol. 2." Liberty Fund.

[ref10] Berger Peter L. Luckmannn Thomas. (1967). The social construction of reality: a treatise in the sociology of knowledge. New York: Doubleday.

[ref11] Bloch Maurice. (1989). Ritual, history and power: selected papers in anthropology. London: Athlone Press.

[ref12] Bourdieu Pierre. (1977). Outline of a theory of practice. Cambridge: Cambridge University Press.

[ref13] Bourdieu Pierre. (1995). The logic of practice. Stanford: Stanford University Press.

[ref14] Braun D. Gilardi F. (2006). Taking ‘Galton's problem’ seriously: towards a theory of policy diffusion. J. Theor. Polit. 18, 298–322. doi: 10.1177/0951629806064351

[ref15] Bromley Patricia Meyer John W. (2015). Hyper-organization: global organizational expansion. Oxford: Oxford University Press.

[ref16] Callon M. (1986). “Some elements of a sociology of translation: domestication of the scallops and the fishermen of St Brieuc Bay” in Power, action, and belief: a new Sciology of knowledge? ed. Law J. (London: Routledge & Kegan Paul), 196–223.

[ref17] Czarniawska B. Sevón G. (1996). “Introduction” in Translating organizational change. eds. Czarniawska B. Sevón G. (Berlin: Walter de Gruyter), 1–12.

[ref18] Drew Paul Heritage John. (1992). Talk at work: interaction in institutional settings. Cambridge: Cambridge University Press.

[ref19] Durkheim Émile. (1995). The elementary forms of religious life. New York: The Free Press.

[ref20] Ekstrom M. (2012). Gaze work in political media interviews. Discourse Commun. 6, 249–271. doi: 10.1177/1750481312452200

[ref21] Ferraris Maurizio. (2013). Documentality: why it is necessary to leave traces. New York: Fordham University Press.

[ref22] Finnish Patent and Registration Office. (2022), Finnish associations act. Available at: https://www.prh.fi/en/yhdistysrekisteri/act.html (Accessed November 20, 2022).

[ref23] Garfinkel H. (1964). Studies of the routine grounds of everyday activities. Soc. Probl. 11, 225–250. doi: 10.1525/sp.1964.11.3.03a00020

[ref24] Garfinkel Harold. (1967). Studies in ethnomethodology. Englewood Cliffs, NJ: Prentice-Hall.

[ref25] Gennep Arnold van. (1960). The rites of passage. Chicago: University of Chicago Press.

[ref26] Goffman E. (1955). On face-work: an analysis of ritual elements in social interaction. Psychiatry 18, 213–231. doi: 10.1080/00332747.1955.11023008 13254953

[ref27] Goffman Erving. (1956). The presentation of self in everyday life. Edinburgh: University of Edinburgh, Social Sciences Research Centre.

[ref28] Goffman Erving. (1974). Frame analysis: an essay on the Organization of the Experience. New York: Harper Colophon.

[ref29] Habermas Jürgen. (1984). The theory of communicative action, volume 1: reason and the rationalization of society. Boston: Beacon Press.

[ref30] Heritage J. (2009). “Conversation analysis as social theory” in The new Blackwell companion to social theory. ed. Turner B. S. (Chichester: Wiley-Blackwell), 300–320.

[ref31] Ilomäki S. Ruusuvuori J. (2022). Preserving client autonomy when guiding medicine taking in Telehomecare: a conversation analytic case study. Nurs. Ethics 29, 719–732. doi: 10.1177/09697330211051004, PMID: 35119321 PMC9127937

[ref32] Islam G. Zyphur M. J. (2009). Rituals in organizations: a review and expansion of current theory. Group Org. Manag. 34, 114–139. doi: 10.1177/1059601108329717

[ref33] Lane Christel. (1981). The rites of rulers: ritual in industrial society — The soviet case. Cambridge: Cambridge University Press.

[ref34] Latour B. (1986). “The powers of association” in Power, action and belief: a new sociology of knowledge? ed. Law J. (London: Routledge), 261–277.

[ref35] Latour B. (1994). On technical mediation - philosophy, sociology, genealogy. Comm. Knowled. 3, 29–62.

[ref36] Meyer J. W. Rowan B. (1977). Institutionalized organizations: formal structure as myth and ceremony. Am. J. Sociol. 83, 340–363. doi: 10.1086/226550

[ref37] Meyer J. W. Boli J. Thomas G. M. Ramirez F. O. (1997). World society and the nation-state. Am. J. Sociol. 103, 144–181. doi: 10.1086/231174

[ref38] Meyer J. W. Bromley P. (2013). The worldwide expansion of “organization”. Soc. Theory 31, 366–389. doi: 10.1177/0735275113513264

[ref39] Meyers R. A. (2016). “Christian marriage and funeral services as rites of passage” in Oxford research encyclopedia of religion. ed. Barton J. (Oxford: Oxford University Press), 1–17.

[ref40] North Douglass C. (1990). Institutions, institutional change, and economic performance. Cambridge: Cambridge University Press.

[ref41] Ozbolat A. (2019). From Ijazah ceremony to graduation Celebration: continuity and change in the ritual of a rite of passage. Millî folklor 2019, 102–114. Available at: https://www.millifolklor.com/PdfViewer.aspx?Sayi=121&Sayfa=104

[ref42] Palonen Kari. (2019). Parliamentary thinking: procedure, rhetoric and time. New York: Palgrave Macmillan.

[ref43] Palonen Kari. (2022). We politicians: translation, rhetoric and conceptual change. Paper presented at the FPSA annual conference, May 12, 2022, Helsinki.

[ref44] Parsons Talcott. (1951). The social system. Glencoe, IL: Free Press.

[ref45] Parsons T. (1964). Evolutionary universals in society. Am. Sociol. Rev. 29, 339–357. doi: 10.2307/2091479

[ref46] Parsons Talcott. (1966). Societies: evolutionary and comparative perspectives. Englewood Cliffs: Prentice-Hall.

[ref47] Pekonen O. (2017). The political transfer of parliamentary concepts and practices in the European periphery: the case of obstruction in late nineteenth- and early twentieth-century Finland. Parl. Est. Rep. 37, 281–300. doi: 10.1080/02606755.2017.1326558

[ref48] Rawls John. (1971). A theory of justice. Cambridge: Belknap Press of Harvard University Press.

[ref49] Ross M. H. Homer E. (1976). Galton's problem in cross-national research. World Polit. 29, 1–28. doi: 10.2307/2010045

[ref50] Strang D. Meyer J. W. (1993). Institutional conditions for diffusion. Theory Soc. 22, 487–511. doi: 10.1007/BF00993595

[ref51] Syväterä J. Qadir A. (2015). The construction and spread of global models: worldwide synchronisation and the rise of National Bioethics Committees. Eur. J. Cult. Polit. Sociol. 2, 267–290. doi: 10.1080/23254823.2016.1147370

[ref52] Taira T. (2010). Religion as a discursive technique: the politics of classifying Wicca. J. Contemp. Relig. 25, 379–394. doi: 10.1080/13537903.2010.516546

[ref53] Taira Teemu. (2022). Taking 'Religion' seriously: essays on the discursive study of religion. Leiden: Brill.

[ref54] Twining W. (2004). Diffusion of Law: a global perspective. J. Legal Plural. Unoff. Law 36, 1–45. doi: 10.1080/07329113.2004.10756300

[ref55] Vieira Ryan A. (2015). Time and politics: parliament and the culture of modernity in Britain and the British world. Oxford, United Kingdom: Oxford University Press.

[ref56] Watson Alan. (1974). Legal transplants: an approach to comparative Law. Charlottesville: University Press of Virginia.

[ref57] Weatherford J. McIver. (1997). The history of money: from sandstone to cyberspace. New York: Crown Publishers.

[ref58] Wikipedia. (2022), Sweat lodge. https://en.wikipedia.org/wiki/Sweat_lodge (Accessed November 20, 2022).

[ref59] Zimmerman D. H. Pollner M. (1970). “The everyday world as a phenomenon” in Understanding everyday life: toward the reconstruction of sociological knowledge. ed. Douglas J. D. (Chicago: Aldine Publishing Company), 80–103.

